# Supplementary figures and images for: Membrane particles from mesenchymal stromal cells reduce the expression of fibrotic markers on pulmonary cells
Source: PLoS One. 2021 Mar 17;16(3):e0248415. doi: 10.1371/journal.pone.0248415 (PMC7968667; doi:10.1371/journal.pone.0248415)

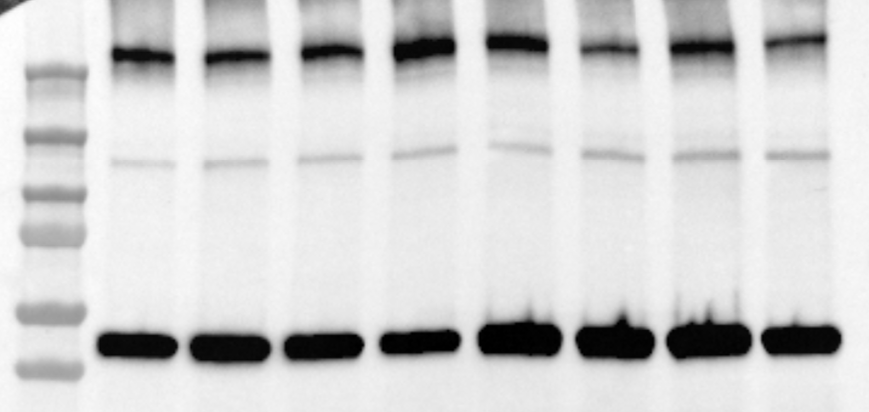

Supplement: S1 Fig — (TIF) [file pone.0248415.s001.tif]
