# Supplementary material for: Membrane particles from mesenchymal stromal cells reduce the expression of fibrotic markers on pulmonary cells
Source: PLoS One. 2021 Mar 17;16(3):e0248415. doi: 10.1371/journal.pone.0248415 (PMC7968667; doi:10.1371/journal.pone.0248415)
Supplement: Attachment — Submitted filename: Response to Reviewers.docx [file pone.0248415.s003.docx]

**Response to the Reviewers:**

Additional Editor Comments (if provided): In the revised manuscript the authors made only small improvements. The answers given by authors to reviewer’s comments are not convincing. Specific comments:

1. Following your reply for Figure 6A and 6B, you did not find any significant between the result. You have to describe the exact statical mean+/-SEM. After that, we will assume your results carefully.

**After a deeper revision of the statistical analysis and discussion with the Statistician of our institution, the Reviewer was right and the results of figures 6A and 6B were significant. We run the Mann Whitney test, and obtained the following mean±SEM data for COL1A1 (6A): TGF-β (5.122 ± 0.3703) vs TGF-β-MP (3.013 ± 0.2569) and for alpha-sma (6B): TGF-β (16.51 ± 1.686) vs TGF-β-MP (10.83 ± 0.9152). In both cases the p value was <0.05.**

**We apologize for missing the correction in the last Revised version of the manuscript, we have modified accordingly the figure by adding the asterisk in the correct columns.**

**We thank the Reviewer´s comment and appreciate the opportunity to correct this mistake.**

2. Answering for reviewer 3 the authors wrote still ‘a potential promising therapy’ in abstract. The results are not supported as a promising therapy.

**As per suggestion, we have changed the conclusion paragraph in the Abstract section**.

3. It is not enough to satisfy for reviewer 3 why a protein-free product would be a goal. If you have any reference, you describe it in the manuscript directly.

**As it is being described by the International Society of Extracellular Vesicles (J Extracell Vesicles. 2018;7(1):1535750), obtaining a pure preparation of EV is still a challenge. During the isolation protocol, soluble proteins co-precipitate with the EV being a confounding factor in the interpretation of the results. It is not possible to know whether the effect of the preparation is due to the EV or the contaminated proteins. This is the reason that a contaminant-protein-free product would be the ideal goal to better know the true effect of EV.**

**In the MP generation protocol, we avoid this co-precipitation of proteins and other soluble factors by discarding the supernatant of the cells.**

**We have added and cited the new reference in the Introduction section.**

4. In materials and methods, you mentioned about the procedure about polycarbonate membrane filter. However, we cannot find any details in your manuscript. Even thought, you used three different size filters, we couldn’t understand well. Did you use them step by step or individually or respectively?

**We gradually pass through different pore size filters the MP, from bigger to smaller size (800-400-200 nm). The sequential use of these filters make it possible to obtain the final desired particle size at 200nm without blockade the filter. This is a method described for liposome preparation (Zhang H. Thin-Film Hydration Followed by Extrusion Method for Liposome Preparation. Methods Mol Biol. 2017;1522:17-22. doi: 10.1007/978-1-4939-6591-5_2. PMID: 27837527).**

**We´ve re-written the methodology section to better clarify this point.**

5. In results, what is the reference values in line #321 in revised manuscript?

**The term *reference values* refers to predicted values of standardization of lung function testing. (Eur Respir J 2005; 26: 319–338 and Eur Respir J 2005; 26: 720–735).**

**We have added these references in the Revised Manuscript.**

6. Answering for reviewer 3’s comment, the author wrote “we have modified this sentence in the Manuscript to avoid the confusion. All the MP found by cryo-EM were lipid bilayer, we sis not observed any single layer” in 2nd sentence in Results ‘characterization of MP generated from AT-MSC’. However, you just removed the 2nd sentence without any modification. Is it your final answering?

**We agree with the Reviewer that we just removed the second sentence because it was confusing but, in our opinion, there is nothing else to add. This sentence, in the previous version, made the reader think that we had both single double phospholipid layers in our sample, and we had only bilayer.**

7. Reviewer 3 mentioned that the author should explain the figure legend for understanding in figure 1B. However, author did not describe anything in here. Even though the reviewer wants to know what the concentration of MP per ml means, there is no description.

**We have modified the legend in figure 1B according to the Reviewer´s comment.**

8. All limitations mentioned by reviewer should be at least shortly described in Discussion section. In the revised manuscript Discussion part was almost the same as in original submission although the reviewer asked for improvements. In addition, the author just omitted the description without addition.

**We thank the Reviewer´s comment and, as per suggestion, we have changed the Discussion section to improve the final version of the Manuscript by adding all the potential limitations and concerns raised by the Reviewers.**
